# Miscellany

**Published:** 1892-01

**Authors:** 


### Miscellany.

We call the attention of our readers to Messrs. John Wyeth & Brother's new advertisement, which appears in this number, relating to their compressed tablets of new remedies (antipyretics) for influenza, neuralgia, headache, etc. Their great convenience for administration commends them to the profession.

---

Always at the Front.—We have received a copy of the handsomely illustrated prospectus for 1892, issued by the *Detroit Free Press.* The achievements of this famous paper in the past have been great, but if its promises for the future are to be fulfilled—and there certainly is no reason to expect the contrary—the *Detroit Free Press* will, in 1892, be, as its publishers confidently claim, the most entertaining and instructive paper published, giving additional pleasure to its thousands of old subscribers, and fresh enjoyment to the many thousand new ones that its merits deserve. Its list of contributors for 1892 includes many of the most famous names in American literary and public life, and most of the articles to be published are of unusual importance and interest, presenting a splendid array of valuable features in addition to the inimitable work done by its own staff of bright and famous writers.

The publishers of *The Free Press* will mail copies of the paper and prospectus to all applicants.

---

Happy and content is a home with "The Rochester; a lamp with the light of the morning. Catalogues, write Rochester Lamp Co., New York.

---

Current Medical Literature.—In the next number of the *Sanitary Era* will be commenced a regular list of the principal contents of the medical journals for the month, which, it is hoped, will be valuable to every physician as well as to professional exchanges themselves. Few can afford, and still fewer could find time to explore, the large number of such periodicals, each of which, from time to time, contains something which the individual practitioner or student of medicine would be very sorry to miss, and yet nearly sure to miss, without such an index as is proposed. Sample copies will be sent on request. The editor would also like to be advised how far an extension of the index to foreign periodicals might probably be encouraged by subscriptions to the paper.

